# Factors associated with the acceptability of self-injection training by clients receiving DMPA-SC services from community pharmacies and patent and proprietary medicine vendors in Nigeria

**DOI:** 10.1186/s12905-025-03945-3

**Published:** 2025-08-21

**Authors:** Toyin O. Akomolafe, Emeka Emmanuel Okafor, Sikiru Baruwa, Osimhen Ubuane, Michael Alagbile, Delafrida Ukaga, Ijeoma Nwankwo, Rodio Diallo

**Affiliations:** 1 Population Council, Abuja, Nigeria; 2 Society for Family Health, Abuja, Nigeria; 3 Pharmaceutical Society of Nigeria Foundation, Abuja, Nigeria; 4 Bill & Melinda Gates Foundation, Abuja, Nigeria

**Keywords:** Family planning, Subcutaneous depot medroxyprogesterone acetate (DMPA-SC), Self-injection, Community pharmacist, Patent and proprietary medicine vendors, Private sector, Quality of care, Acceptability

## Abstract

**Background:**

The self-administration of the subcutaneous depot medroxyprogesterone acetate (DMPA-SC) has the potential to empower women and improve contraceptive use. Several studies have demonstrated the acceptability of the provider-administered DMPA-SC, for private, public, or community-based health providers, but less is known about self-injection training from community pharmacists (CPs) and Patent and Proprietary medicine Vendors (PPMVs) (also known as drug shops). The purpose of this study was to examine the factors associated with the acceptance of DMPA-SC self-injection training when provided by trained CPs and PPMVs in Lagos and Kaduna states.

**Methods:**

A cross-sectional study was conducted among 186 women of reproductive age (18–49 years) who received self-injection training on DMPA-SC from trained CPs and PPMVs between August and September 2019, and between May 2022 and June 2023. Women who selected DMPA-SC, opted for self-injection, and were trained by a CP or a PPMV were invited to participate in the study. Quantitative telephone interviews were conducted with eligible clients within six (6) weeks of obtaining DMPA-SC commodities for self-injection from a trained CP or PPMV. Bivariate chi-square test and multivariate logistic regression were used to examine factors associated with acceptability and continued self-injection of DMPA-SC at home. The results were considered significant at a *p* value < 0.05.

**Results:**

More than half of the women had used a contraceptive in the past (55%), and 73% received information on DMPA-SC from a CP or PPMV. Using a family planning method prior to visiting the provider (Odds ratio (OR) = 4.31; 95% Confidence Intervals (CIs): 1.05, 17.69; p = 0.04), receiving information on DMPA-SC from friends/relative (OR = 5.08; 95% CI: 1.01, 25.62; p = 0.05), perceived high-quality care (OR = 7.72; 95% CI: 2.52, 23.61; *p* = 0.00) and middle-quality care (OR = 3.35; 95% CI: 1.16, 9.69; *p* = 0.03) were significantly associated with the likelihood of continued DMPA-SC self-injection at home. A high level of acceptance of training in DMPA-SC self-injection was significantly associated with service from PPMVs (OR = 4.94; 95% CI = 1.46, 16.75; *p* = 0.01) and perceived high-quality care (OR = 4.23; 95% CI = 1.62, 11.05; *p* = 0.00).

**Conclusions:**

The results are promising for expanding DMPA-SC self-injection service delivery in Nigeria through increased method choice, and empowered users. The provision of counseling and DMPA-SC self-injection training by CPs and PPMVs is acceptable among women in Lagos and Kaduna states.

## Background

Recent estimates have shown that 218 million women of reproductive age (15–49) in low- and middle-income countries have an unmet need for modern contraceptives– meaning that they do not wish to become pregnant but are not using contraception [1]. Nigeria has unmet needs for family planning (FP) among sexually active unmarried women (48%) and married women (19%) [2]. This has led to high population rates, increased unintended pregnancies and increased unsafe abortion [3, 4].

Less than a sixth of the currently married women aged 15–49 years in Nigeria use modern contraceptives (12%), with injectables and implants (3%) being the most used [5]. Other FP methods accounted for 2% or less; pills (1%), male condoms (2%), and Intrauterine devices (IUDs) (1%), this showed that contraceptive uptake by method is very low [5]. Barriers to uptake include long distances to clinics, poor service quality, high workloads at family planning clinics, and general inconvenience. [6–8]. Many women also require frequent visits to healthcare facilities for contraceptive administration, which is particularly challenging in underserved areas [7]. As a result, the private sector has become a reliable option for family planning with, 40% of family planning clients in Nigeria preferred obtaining contraceptives through private sector providers, with more than half (60%) accessing family planning through patent proprietary medicine vendors (PPMVs) [5]. This preference is attributed to the private sector's widespread availability, consistent drug stocks, extended operating hours, and free consultations [9, 10].

Introduction of self-administered contraceptives like subcutaneous depot medroxyprogesterone acetate (DMPA-SC) has the potential to mitigate some of these barriers and increase uptake [11]. The concept of self-administered contraceptives has the potential to empower women to make informed family planning and sexual reproductive decisions [12]. It effectively addresses critical issues such as cost-effectiveness, time and reduced reliance on public health systems which are often susceptible to commodity stockouts, health worker absenteeism, and limited access to family planning services [5, 12]. Research findings showed that self-injection is feasible and acceptable to women [13–15]. It also plays a crucial role in ensuring the uninterrupted use of injectable contraceptives [16]. Depot medroxyprogesterone acetate (DMPA) contains a synthetic hormone called progestin, which mimics the action of natural progesterone. It is available in two delivery forms: the subcutaneous formulation (DMPA-SC), which contains 104 mg of the hormone, and the intramuscular formulation (DMPA-IM), which contains 150 mg. Despite the variation in dosage and route of administration, both formulations have been demonstrated to be safe and effective [11, 17]. DMPA-SC is a self-administered injectable contraceptive that can be administered subcutaneously rather than intramuscularly [17]. This innovation allows women to take control of their contraceptive needs by administering the injections themselves, reducing the need for frequent healthcare visits, and increasing accessibility [11, 17, 18].

In 2014, the subcutaneous depot medroxyprogesterone acetate (DMPA-SC) was introduced in various African countries across the health sectors including community health workers [11–22]. Implementation results showed that it has the capacity to reach more women including adolescents and young adults who are often neglected [11]. A study of self-injection in Uganda showed that nearly 90% of women aged 18–49 years were capable of proficiently self-injecting DMPA-SC three months after a single one-on-one training session [13]. In Nigeria, DMPA-SC was introduced through the private sector in 2015, and later to the public sector in 2016 [23, 24]. Convenience, familiarity with provider and high quality of care were factors associated use of DMPA-SC among clients patronizing private providers [24]. However, the unique selling point of DMPA-SC which is self-injection was not readily accepted by both providers and clients in Nigeria [25, 26]. Providers are of the opinion that contact with clients during use is crucial to address side effects [25]. Among clients, there is preference for provider administration of DMPA-SC due to fear of needles, and lack of confidence in their ability to administer it correctly after training [21, 26]. Most studies on acceptability among clients are focused on the product, that is, DMPA-SC, not the training received by any type of provider [13, 18–21, 27, 28].

To the best of our knowledge, no published studies have explored the client acceptability of DMPA-SC self-injection training from community pharmacists (CPs) or PPMVs in Nigeria. The goal of this study was to examine factors associated with the acceptance of DMPA-SC self-injection training when provided by trained CPs and PPMVs. In this study, we describe the results of quantitative interviews conducted with clients who visited CPs and PPMVs for DMPA-SC self-injection to determine factors associated with their acceptance of training and continued self-injection at home. The results will offer important practical recommendations for informing self-injection accessibility and future scale-up efforts in Nigeria and Africa.

## Methods

### Intervention

In 2017, the IntegratE project funded by the Bill & Melinda Gates Foundation and Merck Sharp & Dohme for Mothers commenced implementation in Kaduna and Lagos states in Nigeria. In collaboration with the Pharmacy Council of Nigeria (PCN), the IntegratE Project aims to increase access to family planning services and contraceptive methods in underserved areas through the involvement of private sector providers, CPs and PPMVs. The project currently covers eleven states in Nigeria and, in addition to family planning, layered a modicum of primary health care services. A core delivery vehicle for the implementation of family planning services is adopting the PCN three-tier approach. PCN classified PPMVs into three groups or tiers, and different scopes of family planning training were provided based on their health qualifications. The three tiers are: Tier 1 (nonhealth trained), Tier 2 (health trained and Tier 3 (pharmacy technicians). A detailed description of the three-tier accreditation system of the IntegratE Project, the categories of PPMVs and the type of training received can be found elsewhere [29, 30].

Under the IntegratE Project, CPs and health-trained PPMVs (Tiers 2 and 3) were trained to provide information on all modern contraceptive methods, including DMPA-SC self-injection training, to all female FP clients visiting the PPMVs. Specifically, on the self-injection, the CPs and PPMVs were trained on how to train clients on the following aspects of the DMPA-SC: (1) self-injection technique; (2) mixing of solution; (3) activation of device; (4) appropriate injection sites; (5) safe storage; (6) safe disposal of used devices in a puncture-proof container; and (7) calculation of reinjection dates. Training included a review of DMPA side effects and protection against HIV (Human Immunodeficiency Virus), and appropriate disposal practices (e.g., returning used syringes in puncture-proof containers to the provider for appropriate biological waste disposal). The trained CPs and PPMVs counseled DMPA-SC clients who chose to self-inject and trained them as above and allowed women to self-inject under the supervision of the providers. Clients who self-injected at the provider facility were given up to 2 DMPA-SC doses for home self-injection, along with a self-injection instruction sheet, a puncture-proof container, and a reinjection calendar with dates for the next two injections circled.

### Study settings

This study took place in the two IntegratE Phase 1 states of Lagos and Kaduna. Since 2017, when the Society for Family Health (SFH) starts implementing the project in these states, providers have become more established in their role of providing expanded family planning services. According to the 2018 NDHS, 14% and 29% of married women in Kaduna and Lagos respectively, were using modern contraceptives [5]. In Lagos, women had a high desire to limit childbearing (37%) and 17% had an unmet need for FP. Kaduna has one of the highest fertility rates in Nigeria with a low desire to limit childbearing (16%), and 12% of these women have an unmet need for FP [5].

### Study design/sampling and recruitment procedures

A cross-sectional study was conducted to assess clients’ experiences with DMPA-SC self-injection services from IntegratE trained CPs and PPMVs in Lagos and Kaduna states. The data collection was conducted between August and September 2019 and between May 2022 and June 2023. Quantitative telephone interviews were conducted with eligible clients within six (6) weeks of obtaining DMPA-SC commodities for self-injection from trained CPs or PPMVs. Women who selected the DMPA-SC, opted for self-injection, and were trained by CP or PPMV were recruited to participate in the study about their experience. All women aged 18—49 years were invited to participate in the survey if they opted for self-injection and were trained by CPs and PPMVs trained by the project in the two states. CPs and PPMVs were asked to inform their clients who were trained on self-injection of DMPA-SC about the study and requested their permission to share their details, including phone numbers, with the IntegratE Research, Monitoring and Evaluation (RME) team. During the data collection period, all 590 women who sought self-injection services from trained CPs and PPMVs were contacted. Of these, 186 new and returning DMPA-SC users who reported self-injecting provided informed consent and were interviewed.

### Data collection

The data were collected from each study participant during phone interviews using a structured questionnaire. Four interviewers participated in training on research ethics, the study’s design and objectives, procedures for providing informed consent forms, and telephone interviews. Trained data collectors contacted eligible women aged 18 to 49 years who consented to participate and provided phone numbers; explained the study details, including the study objectives; what was expected of them as respondents; and potential risks and benefits to participating and thereafter administering the survey. Phone interviews were conducted in English, Yoruba or Hausa language as preferred by participants, and the data were entered using devices preprogrammed with the OpenDataKit (ODK) application. To prevent missing data, checks were incorporated into the ODK app to ensure all essential fields were completed. These checks prevent incomplete data from being submitted. The questionnaire was used to collect information on participants’ background characteristics, experience with family planning, quality of care received (using the validated 22-item process quality measure), and use of tools to support home self-injection. A survey tool from a previous study with face and content validity established was adapted and used [31]. The completed questionnaires in the ODK app were synched to the KoboToolbox online server daily, which the study coordinator reviewed for quality. Respondents received compensation for mobile phone credits worth one thousand Naira (equivalent to $1.50) for their participation in this study. Ethical approval was received from the Population Council's Institutional Review Board (Protocol # 878), and the National Health Research Ethics Committee (NHREC) of the Federal Ministry of Health (NHREC/01/01/2007–16/04/2018).

### Measures

The primary outcome was to assess the acceptability of self-injection training from CPs and PPMVs based on clients’ intention to recommend self-injection to a friend, recommend provider seen to a friend, intention to continue self-injection at home and satisfaction with self-injection training. The questions asked were as follows: (1) “Would you recommend self-injection to a friend? (2) Would you recommend the CP or PPMV you saw to a friend interested in self-injection? (3) Do you plan to continue self-injection at home? (4) How satisfied were you with the self-injection information (and services) received from the CP or PPMV?” The response options for the first three questions were binary (i.e., “yes” or “no”), while the fourth question which was nonbinary (highly satisfied, satisfied, somewhat satisfied, somewhat unsatisfied or not at all satisfied), was categorized as a binary variable. Responses were dichotomized as “yes, satisfied (highly satisfied and satisfied) or not satisfied (somewhat satisfied, somewhat unsatisfied, not satisfied at all). An acceptability score was generated using these four (4) items. The median score was used as the cutoff for “high acceptability” versus “low acceptability”, because it is a more representative measure of central tendency and data is not normally distributed [32]. The secondary outcome was the continuity of DMPA-SC self-injection by women, which is a predictor of acceptance. The “continued DMPA-SC self-injection at home”, as a dependent variable, was also used to measure satisfaction with DMPA-SC self-injection training by CP or PPMV.

**Fig. 1:**
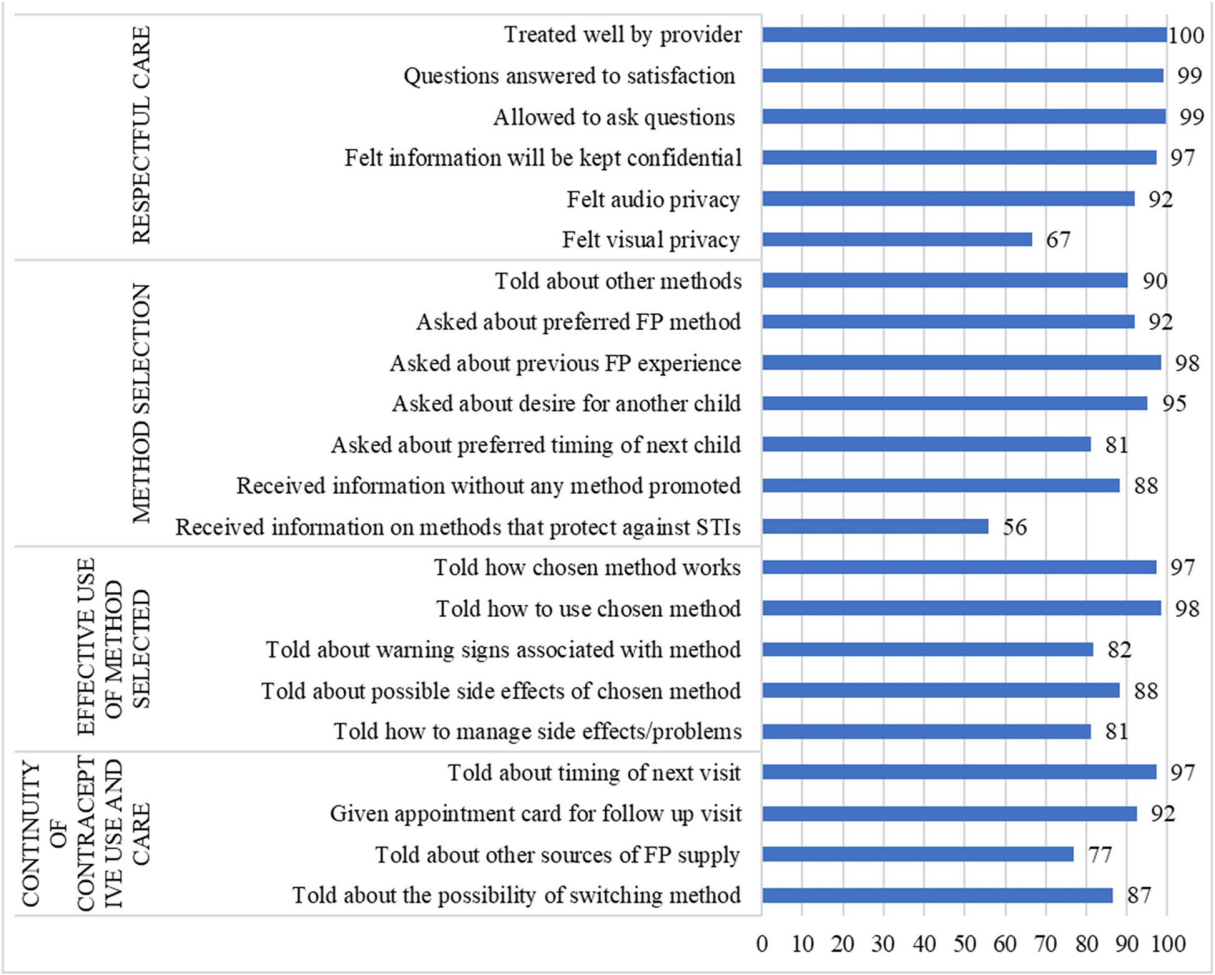
Quality items (*n* = 186)

The independent variables of interest were age (grouped as ≤ 25 years and > 25 years), marital status (not married or married/in union), number of living children, level of education (no formal education, primary, secondary school, and 2 years postsecondary), type of provider seen (CP and Tier 2 PPMV), self-injection experience (first-time and previous self-injection users), state, previous use of FP, FP use prior to visit, wanting more children (within 24 months, > 24 months, undecided, and don’t want), source of information on DMPA-Sc, experienced side effects, and quality of care. Quality of care was measured based on the four domains of the Bruce-Jain framework comprising twenty-two items which were validated [33, 34]. The items for each domain, respectful care (6 items), method selection (7 items), effective use of method selected (5 items), and continuity of contraceptive use and care (4 items), were measured using binary response options (i.e., “yes” or “no”). Figure 1 shows the proportion of respondents with positive responses for each item by domain. A weighted additive quality score was calculated for each woman by computing the average score by domain, multiplying the domain averages by 100 and dividing by the total number of domains. The quality-of-care score which ranges from 0 to 100 was grouped into three categories: low, medium, and high-quality scores [34, 35]. The low quality score ranged from zero to average score minus one-half of standard deviation and the high-quality score was greater than or equal to mean score plus one-half of standard deviation [35].

### Data analysis

We conducted bivariate chi-square tests to examine acceptability and continued DMPA-SC self-injection at home by independent variables (background characteristics, FP experience, fertility intentions, and quality of care). We used multivariate logistic regression models to assess the associations between acceptance of the training, continuation of self-injections at home and statistically significant independent factors according to the bivariate analysis and those of theoretical importance. We included age in the models for quality of care to adjust for confounding variables. To account for potential confounding, age and education were included as covariates in the regression models. We presented the results of the regression analyses as odds ratios (OR) and 95% confidence intervals (95% CI) for all regression models. We conducted all analyses using Stata version 15 (Statacorp). We considered a *p* value < 0.05 to indicate statistical significance.

## Results

Table 1 presents the characteristics of the respondents. Approximately one-quarter of women were aged 25 years or younger (26%). Almost all the respondents were married or in unions (94%) and most had two or more children (80%). Most respondents attended secondary school/postsecondary school (84%). More than half of the women had used a contraceptive method in the past (55%), 73% received information on DMPA-SC from a CP or PPMV, and nearly half (49%) reported receiving high quality care at their visit with the provider. Figure 1 shows the 22 items included in the quality score. Most of the quality items were high (more than 75%), except for two items: one in the respectful care domain and one in the method selection domain. In the respectful care domain, sixty-seven percent of the clients reported that they experienced visual privacy. In the method selection domain, most of the women reported that they were asked about previous FP experience (98%), desire for another child (95%) and their preferred FP method (92%). More than half of the women (56%) reported that they received information on methods that protect against Sexually transmitted infections (STIs). Women reported receiving information about the effective use of the method selected, with most told how the chosen method works (97%), how to use the method (98%) and, more than three-quarter were told about possible side effects (88%) and how to manage them (81%). Almost all the women were told about the timing of next visit (97%), whereas 77% were told about other sources of FP supply.

**Table 1:** Sociodemographic variables and fertility intentions of women who opted for self-injection of the DMPA-SC (*n* = 186)

Client characteristics	N	%
Age		
≤ 25 years	49	26
> 25 years	137	74
Marital Status		
Not married	12	6
Married/In-Union	174	94
Number of living children		
Zero– One	37	20
Two	37	20
Three	65	35
Four or More	47	25
Highest level of education achieved		
No formal education or primary school	29	16
Secondary	88	47
2 + years postsecondary	69	37
Cadre of Provider Seen		
Tier 2 PPMV	156	84
CP	30	16
Self-injection experience		
First-time SI users	127	68
Previous SI users	59	32
State		
Kaduna	111	60
Lagos	75	40
FP Experience/Fertility Intentions		
Ever Used FP		
Yes	102	55
FP Method Previously Used		
Injectables	54	29
Implants	31	17
Oral Pills	23	12
IUD	9	5
Male Condoms	6	3
Using FP prior to visit		
Yes	42	23
Method Using before visit		
Injectables	25	14
Others	17	9
None	144	77
Want More Children		
Within 24 months	45	24
> 24 months	51	28
Undecided	10	5
Don’t Want	80	43
Source of Information on Sayana Press		
CP/PPMV	136	73
Friends/Relatives	36	19
Knew CP/PPMV sold Sayana Press before visit		
Yes	47	25
Quality of care		
Low	44	24
Middle	50	27
High	92	49
Side effects/problems as a result of self-injection		
Experienced side effects	16	9
No side effects	170	91

Tables 2 and 3 show the associations between continued DMPA-SC self-injection at home, as a measure of satisfaction with DMPA-SC self-injection training by a CP or PPMV, and client characteristics. The intention to continue DMPA-SC self-injection and the acceptability of the training received were significantly associated with the type of providers visited for self-injection training and services. Ten percent of those who intend to continue self-injection at home visited a CP, whereas a higher percentage (90%) consulted a health-trained PPMV. Likewise, 11% of clients who reported high acceptability of self-injection training visited a CP and 89% visited health-trained PPMV clients. The acceptability of training received and continued self-injection of DMPA-SCs at home for clients in Kaduna (68% and 66%) was greater than that for clients from Lagos State (32% and 34%) (*p* = 0.00). The intention to continue DMPA-SC self-injection at home was associated with previous implant users, those using a method prior to visiting a provider, and those who did not experience side effects/problems because of self-injection. A high quality of care (54%) (*p* = 0.00) was significantly associated with continuing self-injections at home and accepting training.

**Table 2:** Acceptability of training and continued self-injection of DMPA-SC at home by client characteristics (*n* = 186)

Client characteristics	Plans to continue self-injection at home n (%)		*p* value	High acceptancen (%)	Low acceptance n (%)	*p* value
	Yes	No				
	*n* = 159	*n* = 27		*n* = 151	*n* = 35	
Age			0.32			0.07
≤ 25 years	44 (28)	5 (19)		44 (29)	5 (14)	
> 25 years	115 (72)	22 (81)		107 (71)	30 (86)	
Marital Status			0.14			0.08
Not married	12 (8)	0 (0)		12 (8)	0 (0)	
Married/In-Union	147 (92)	27 (100)		139 (92)	35 (100)	
Number of living children			0.99			0.80
Zero—One	32 (20)	5 (18)		31 (20)	6 (17)	
Two	31 (20)	6 (22)		31 (20)	6 (17)	
Three	56 (35)	9 (33)		53 (35)	12 (34)	
Four or More	40 (25)	7 (26)		36 (24)	11 (31)	
Highest level of education achieved			0.68			0.64
No formal education or primary	25 (16)	4 (15)		25 (16)	4 (11)	
Secondary	77 (48)	11 (41)		72 (48)	16 (46)	
2 + years postsecondary	57 (36)	12 (44)		54 (36)	15 (43)	
Cadre of Provider Seen			**0.00**			**0.00**
Tier 2 PPMV	143 (90)	13 (48)		135 (89)	21 (60)	
CP	16 (10)	14 (52)		16 (11)	14 (40)	
Self-injection experience			0.80			0.97
First-time SI users	108 (68)	19 (70)		103 (68)	24 (69)	
Previous SI users	51 (32)	8 (30)		48 (32)	11 (31)	
State			**0.00**			**0.00**
Kaduna	108 (68)	3 (11)		100 (66)	11 (31)	
Lagos	51 (32)	24 (89)		51 (34)	24 (69)

**Table 3:** Acceptability of training and continued DMPA-SC self-injection at home by the FP experience/fertility intentions

FP experience/fertility intentions	Plans to continue self-injection at home n (%)		*p* value	High acceptancen (%)	Low acceptancen (%)	*p* value
	Yes	No				
	*n* = 159	*n* = 27		*n* = 151	*n* = 35	
Ever Used FP						
Yes	85 (53)	10 (37)	0.36	80 (53)	22 (63)	0.29
FP Method Previously Used						
Injectables	42 (26)	12 (44)	0.06	42 (28)	12 (34)	0.45
Implants	31 (19)	0 (0)	**0.01**	28 (18)	3 (9)	0.15
Oral Pills	20 (13)	3 (11)	0.83	19 (13)	4 (11)	0.85
IUD	8 (5)	1 (4)	0.77	7 (5)	2 (6)	0.79
Male Condoms	4 (2)	2 (7)	0.18	4 (3)	2 (6)	0.35
Using FP prior to visit						
Yes	35 (22)	7 (26)	0.65	32 (21)	10 (29)	0.35
Method Using before visit			**0.04**			0.45
Injectables	18 (11)	7 (26)		18 (12)	7 (20)	
Others	17 (11)	0 (0)		14 (9)	3 (9)	
None	124 (78)	20 (74)		119 (79)	25 (71)	
Want More Children			0.49			0.43
Within 24 months	40 (25)	5 (19)		39 (26)	6 (17)	
> 24 months	44 (28)	7 (26)		43 (28)	8 (23)	
Undecided	7 (4)	3 (11)		7 (5)	3 (9)	
Don’t Want	68 (43)	12 (44)		62 (41)	18 (51)	
Source of Information on Sayana Press						
CP/PPMV	119 (75)	17 (63)	0.20	113 (75)	23 (66)	0.27
Friends/Relatives	32 (20)	4 (15)	0.52	30 (20)	6 (17)	0.71
Knew CP/PPMV sold Sayana Press before visit						
Yes	41 (26)	6 (22)	0.69	38 (25)	9 (26)	0.95
Quality of care			**0.00**			**0.01**
Low	30 (19)	14 (52)		29 (19)	15 (43)	
Middle	43 (27)	7 (26)		41 (27)	9 (26)	
High	86 (54)	6 (22)		81 (54)	11 (31)	
Side effects/problems as a result of self-injection			**0.05**			0.18
Experienced side effects	11 (7)	5 (19)		11 (7)	5 (14)	
No side effects	148 (93)	22 (81)		140 (93)	30 (86)	

Multivariate regression models of the likelihood of continued self-injection of DMPA-SC at home and acceptability of training received are presented in Tables 4 and 5. Several factors remained significant in the models. The odds of women continuing DMPA-SC self-injection at home was six times greater among those who received services from PPMV (Odds ratio (OR) = 6.04; 95% CI = 1.54, 23.72; *p* = 0.01), and clients living in Kaduna were more likely to continue than those living in Lagos (OR = 14.31; 95% CI = 2.77, 73.88; *p* = 0.00). Using a family planning method prior to visiting the provider (OR = 4.31; 95% CI = 1.05, 17.6; *p* = 0.04), receiving information on DMPA-SC from friends/relatives (OR = 5.08; 95% CI = 1.01, 25.62; *p* = 0.05), perceived high-quality care (OR = 7.72; 95% CI = 2.52, 23.61; *p* = 0.00) and middle quality care (OR = 3.35; 95% CI = 1.16, 9.69; *p* = 0.03) were significantly associated with the likelihood of continued DMPA-SC self-injection at home (Tables 4 and 5).

**Table 4:** Logistic regressions of clients’ characteristics on continued DMPA-SC self-injection and acceptance of training

	Continued DMPA-SC self-injection at home	*p* value	Acceptability	*p* value
	Odds Ratio (95% CI)		Odds Ratio (95% CI)	
Age				
≤ 25 years	Ref		Ref	
> 25 years	0.49 (0.07, 3.26)	0.46	0.36 (0.08, 1.59)	0.18
Number of living children				
Zero or one	Ref		Ref	
Two	4..16 (0.46, 37.91)	0.21	3.93 (0.72, 21.48)	0.11
Three	9.94 (0.74, 134.4)	0.08	5.62 (0.83, 38.08)	0.08
Four or more	2.35 (0.18, 30.29)	0.51	2.39 (0.33, 17.11)	0.38
Highest level of education achieved				
No formal education or primary	Ref		Ref	
Secondary	0.58 (0.13, 2.66)	0.49	0.47 (0.12, 1.82)	0.27
2 + years postsecondary	0.30 (0.06, 1.49)	0.14	0.43 (0.11, 1.74)	0.24
Cadre of Provider Seen				
CP	Ref		Ref	
Tier 2 PPMV	6.04 (1.54, 23.72)	**0.01**	4.94 (1.46, 16.75)	**0.01**
Self-injection experience				
First-time SI users	Ref		Ref	
Previous SI users	0.53 (0.15, 1.86)	0.32	0.74 (0.28, 1.95)	0.54
State				
Lagos	Ref		Ref	
Kaduna	14.31 (2.77, 73.88)	**0.00**	2.56 (0.78, 8.34)	0.12
Ever Used FP				
No	Ref		Ref	
Yes	0.46 (0.12, 1.77)	0.26	0.67 (0.23, 1.93)	0.45
Using FP prior to visit				
No	Ref		Ref	
Yes	4.31 (1.05, 17.69)	**0.04**	1.67 (0.55, 5.14)	0.37
Want More Children				
Don’t Want	Ref		Ref	
Undecided	0.24 (0.03, 2.06)	0.19	0.61 (0.09, 4.00)	0.60
Within 24 months	0.66 (0.09, 4.64)	0.67	1.37 (0.29, 6.41)	0.69
> 24 months	0.92 (0.20, 4.25)	0.91	1.32 (0.38, 4.55)	0.66
Received information from CP/PPMV				
No	Ref		Ref	
Yes	2.80 (0.68, 11.49)	0.15	2.15 (0.65, 7.05)	0.21
Received information from friends/relative				
No	Ref		Ref	
Yes	5.08 (1.01, 25.62)	**0.05**	2.31 (0.66, 8.12)	0.19
Knew CP/PPMV sold Sayana Press before visit				
No	Ref		Ref	
Yes	1.32 (0.35, 5.04)	0.68	1.04 (0.35, 3.12)	0.94
Side effects/problems because of self-injection				
Experienced side effects	Ref		Ref	
No side effects	1.35 (0.26, 7.00)	0.72	0.99 (0.24, 4.10)	0.99

**Table 5:** Logistic regressions of quality on continued DMPA-SC self-injection and acceptability

	Continued DMPA-SC self-injection at home	*p* value	Acceptability	*p* value
	Odds ratio (95% CI)		Odds ratio (95% CI)	
Quality of care				
Low	Ref		Ref	
Middle	3.35 (1.16, 9.69)	**0.03**	2.92 (1.07, 7.98)	**0.04**
High	7.72 (2.52, 23.61)	**0.00**	4.23 (1.62, 11.05)	**0.00**

Models adjusted for age and education

A high level of acceptance of training received in DMPA-SC self-injection was significantly associated with service from PPMVs (OR = 4.94; 95% CI = 1.46, 16.75; *p* = 0.01), perceived high-quality care (OR = 4.23; 95% CI = 1.62, 11.05; *p* = 0.00) and middle quality care (OR = 2.92; 95% CI = 1.07, 7.98; *p* = 0.04) (Table 5).

## Discussion

The aim of this study was to examine factors associated with the acceptance of DMPA-SC self-injection training when provided by community pharmacists (CPs) and patent and proprietary medicine vendors (PPMVs) in Lagos and Kaduna states. Additionally, we examined the characteristics of women associated with continued self-injection of DMPA-SC at home. The findings of this study are one of the few studies investigating DMPA-SC self-injection in the private sector and contributed to evidence on the acceptability of self-injection training in general [13, 21, 22, 36].

Our findings showed that the type of provider visited is associated with continuing DMPA-SC self-injection at home, with higher odds when a health trained PPMV was visited. Health trained PPMVs are usually nurses, midwives, community health extension workers (CHEWs) or community health officers (CHOs), mostly female, and are more likely to have worked in a health facility, though not necessarily in the family planning department [30, 37, 38]. They have high knowledge of injectable contraceptives and are in frequent demand for the administration of injectables, including DMPA-SC [37, 38]. In general, PPMVs are easily accessible within the community, and offer privacy and confidentiality to women [39]. These characteristics may have influenced women to gravitate to them for service provision. The popular patronage suggests the need to ensure that health-trained PPMVs are adequately trained to provide DMPA-SCs and other modern contraceptives. These finding also suggest the need for repeated training and support for CPs who are not traditionally trained to provide similar injectable services.

Women’s decision to continue DMPA-SC self-injection at home was significantly associated with receiving information on the method from friends/relatives. This finding aligns with a similar study in Nepal that examined the acceptability of DMPA-SC. Information on DMPA-SC from close relatives (sisters and other family members), friends or neighbors with previous experience with the method inspires trust and increases the possibility of continuation [40]. Familial support should be promoted through awareness creation to increase self-injection continuation [41].

Clients’ perceptions of quality of care are associated with acceptance of self-injection training received from CPs and PPMVs. This association was also observed with continued self-injection of DMPA-SCs at home, which suggested that the quality of information and care received play key roles in women’s decisions regarding continued self-injection. The likelihood of continuing with self-injection at home is higher when women report experiencing moderate or high-quality care compared to when they receive care of low quality. This shows that an improvement in the quality of care may result in an increased intention to continue the method at home. These findings were related to a study conducted in the southwestern states of Nigeria, which showed that high-quality counseling and the absence of side effects increased the likelihood of continued use of the DMPA-SC provided by public health providers [42]. However, our study showed that the absence or presence of side effects was not significantly associated with the acceptance of self-injection training or continued self-injection of DMPA-SCs at home. In this study, the proportion of women who reported visual privacy was the lowest in the respectful care domain, and information on methods that protect against STIs was the lowest. These findings are similar to those of previous studies among the same population of women visiting CPs and PPMVs [30] and suggest that there is a need for improvement in training and support provided to these providers.

Women using the family planning method prior to visiting a CP or PPMV were more likely to continue DMPA-SC self-injection at home than women who were not using it. This may be due to the existing desire to limit childbearing or positive experience using contraceptives. Prior contraceptive use has been shown to be a strong predictor of intending to continue use [43, 44]. This contradicts the findings of a previous study that showed that first-time FP use was associated with continued DMPA-SC use through self-injection [15]. Marital status and education are not predictors of continued self-injection of the DMPA-SC at home. These findings differ from those of previous studies [15] and may be due to the context of intervention implementation.

The findings of this study are important for the following reasons. This study provides evidence that CPs and PPMVs can be trained to provide quality family planning services, including training on DMPA-SC self-injection. The acceptance of their role in the provision of injectable contraceptives, including the self-injection option of the DMPA-SC, may improve access to family planning and potentially impact modern contraceptive use. Our findings showed that women can be trained successfully by CPs and PPMVs to self-inject DMPA-SCs at home. Nevertheless, there is room for improvement in the quality of care received from these providers, which may affect continued use. However, program for DMPCA-SC self-injection may be made more effective with appropriate information, education and communication materials disseminated to women and community members as continuation of DMPA-SC SI at home was significantly associated with receiving information from friends and relatives.

This study has several limitations. The clients interviewed may have slight recall issues, as interviews were conducted between two and six weeks after visiting to trained CPs and PPMVs. Women who participated in this study were not randomly selected; they were clients who visited CPs and PPMVs on the IntegratE project, agreed to be contacted, and provide a phone number for follow-up interview. Providing a valid phone number could introduce selection bias. Additionally, we did not observe self-injection or 3-month reinjection time directly, and it is possible that the self-injections reported may not have mirrored women's actual practices. Therefore, the findings from this study may not be generalizable to all DMPA-SC clients in Lagos and Kaduna, nor other parts of Nigeria. The study was not conducted to test any hypothesis, it was program implementation and to add new knowledge on the research problem. Due to the small sample size of the study, generalization to DMPA-SC clients patronizing CPs and PPMVs is limited. Lastly, while this study assessed acceptability of clients’ self-injection training and the intention to continue with SI at home, there is a need for more studies on other critical factors ("fear of the needle", user's skill proficiency to safely SI, availability of take home-doses, and"covert use") that drive SI practices at home.

## Conclusion

This study adds to the growing body of literature exploring the provision of self-injection training by nonformal health workers and is the first to examine the acceptability of training on DMPA-SC self-injection by CPs and PPMVs in the private sector. The women in this study found self-injection training acceptable, when provided by health-trained PPMVs and perception of quality of care was medium or high. It also highlights the role of health-trained PPMVs, prior FP use, perceived quality of care, and social networks on continued self-injection of DMPA-SC at home. Overall, the findings showed the need for training and retraining CPs and PPMVs to provide self-injection services. The results are promising for expanding DMPA-SC self-injection service delivery in Nigeria by increasing method choice and empowering users.

## Data Availability

The deidentified dataset analyzed during the current study will be available on the Population Council’s Dataverse, https://doi.org/10.7910/DVN/QI0VBT. The data will also be available from the corresponding author upon reasonable request.

## Abbreviations

CHEWs: Community Health Extension Workers
CHOs: Community Health Officers
CPs: Community Pharmacists
DMPA-SC: Subcutaneous Depot Medroxyprogesterone Acetate
DMPA-IM: Intramuscular Depot Medroxyprogesterone Acetate
FP: Family Planning
LMIC: Low- and Middle-Income Country
NDHS: National Demographic Health Survey
OR: Odds Ratio
PCN: Pharmacy Council of Nigeria
PPMVs: Patent and Proprietary Medicine Vendors
RME: Research, Monitoring and Evaluation
SI: Self-Injection
STIs: Sexually transmitted infections
